# Genetic polymorphisms in the circumsporozoite protein of *Plasmodium malariae* show a geographical bias

**DOI:** 10.1186/s12936-018-2413-3

**Published:** 2018-07-16

**Authors:** Naowarat Saralamba, Mayfong Mayxay, Paul N. Newton, Frank Smithuis, Francois Nosten, Laypaw Archasuksan, Sasithon Pukrittayakamee, Nicholas J. White, Nicholas P. J. Day, Arjen M. Dondorp, Mallika Imwong

**Affiliations:** 1 Department of Molecular Tropical Medicine and Genetics, Faculty of Tropical Medicine, Mahidol University, Bangkok, 10400 Thailand; 2 Mahidol Oxford Tropical Medicine Research Unit, Faculty of Tropical Medicine, Mahidol University, Bangkok, Thailand; 3 Lao-Oxford-Mahosot Hospital-Wellcome Trust Research Unit (LOMWRU), Microbiology Laboratory, Mahosot Hospital, Vientiane, Lao PDR; 4 Faculty of Postgraduate Studies, University of Health Sciences, Vientiane, Lao PDR; 5 Centre for Tropical Medicine and Global Health, Churchill Hospital, University of Oxford, Oxford, UK; 6 Medical Action Myanmar, Yangon, Myanmar; 7 Centre for Tropical Medicine, Nuffield Department of Clinical Medicine, University of Oxford, Oxford, OX3 7LF UK; 8 Shoklo Malaria Research Unit, Mahidol-Oxford Tropical Medicine Research Unit, Bangkok, Thailand; 9 Department of Clinical Tropical Medicine, Faculty of Tropical Medicine, Mahidol University, Bangkok, Thailand

**Keywords:** Malaria, *Plasmodium malariae*, Circumsporozoite protein

## Abstract

**Background:**

*Plasmodium malariae* is characterized by its long asymptomatic persistence in the human host. The epidemiology of *P. malariae* is incompletely understood and is hampered by the limited knowledge of genetic polymorphisms. Previous reports from Africa have shown heterogeneity within the *P. malariae circumsporozoite protein* (*pmcsp*) gene. However, comparative studies from Asian countries are lacking. Here, the genetic polymorphisms in *pmcsp* of Asian isolates have been characterized.

**Methods:**

Blood samples from 89 symptomatic *P. malariae*-infected patients were collected, from Thailand (*n* = 43), Myanmar (*n* = 40), Lao PDR (*n* = 5), and Bangladesh (*n* = 1). *pmcsp* was amplified using semi-nested PCR before sequencing. The resulting 89 *pmcsp* sequences were analysed together with 58 previously published *pmcsp* sequences representing African countries using BioEdit, MEGA6, and DnaSP.

**Results:**

Polymorphisms identified in *pmcsp* were grouped into 3 populations: Thailand, Myanmar, and Kenya. The nucleotide diversity and the ratio of nonsynonymous to synonymous substitutions (dN/dS) in Thailand and Myanmar were higher compared with that in Kenya. Phylogenetic analysis showed clustering of *pmcsp* sequences according to the origin of isolates (Asia vs. Africa). High genetic differentiation (Fst = 0.404) was observed between *P. malariae* isolates from Asian and African countries. Sequence analysis of *pmcsp* showed the presence of tetrapeptide repeat units of NAAG, NDAG, and NAPG in the central repeat region of the gene. *Plasmodium malariae* isolates from Asian countries carried fewer copies of NAAG compared with that from African countries. The NAPG repeat was only observed in Asian isolates. Additional analysis of 2 T-cell epitopes, Th2R and Th3R, showed limited heterogeneity in *P. malariae* populations.

**Conclusions:**

This study provides valuable information on the genetic polymorphisms in *pmcsp* isolates from Asia and advances our understanding of *P. malariae* population in Asia and Africa. Polymorphisms in the central repeat region of *pmcsp* showed association with the geographical origin of *P. malariae* isolates and can be potentially used as a marker for genetic epidemiology of *P. malariae* population.

**Electronic supplementary material:**

The online version of this article (10.1186/s12936-018-2413-3) contains supplementary material, which is available to authorized users.

## Background

*Plasmodium malariae* is one of six *Plasmodium* spp. that cause malaria in humans (*Plasmodium falciparum*, *Plasmodium vivax*, *P. malariae, Plasmodium ovale curtisi*, *P. ovale wallikeri* [1], and *Plasmodium knowlesi* [2]). *Plasmodium malariae* exhibits unique characteristics; it is the only *Plasmodium* species with a 72-h long erythrocytic stage in humans, and it can maintain low parasitaemia in humans for a decade [3], while still being infectious to *Anopheles* mosquito (vector). Although *P. malariae* is widely distributed in malaria endemic regions, fewer molecular studies have been conducted in this species compared with those in *P. falciparum* and *P. vivax*. *Plasmodium malariae* often maintains low parasitaemia and commonly co-infects with the highly prevalent species, *P. falciparum* and *P. vivax*. Consequently, designing experiments to study the epidemiology of *P. malariae* is difficult.

Comparative genomics of *Plasmodium* spp., including *P. malariae*, has been used to elucidate the evolutionary history of *Plasmodium* spp. that infect humans [4,5]. Population genetic studies of *P. malariae* should be conducted for more understanding in genetic diversity of this parasite. Measurement of gene polymorphism might be helpful for more understanding in biology of *P. malariae*. The polymorphic genes such as genes encoding for antigen are usually selected for using as genetic markers. One of the prominent surface antigens that is important for sporozoite function and invasion to hepatocyte is the circumsporozoite protein (CSP). CSP has been used as a marker to measure the population diversity of *P. falciparum* [6], *P. vivax* [7], and *P. knowlesi* [8].

CSP is the major surface protein of *Plasmodium* sporozoites. The gene encoding for CSP (*csp*) comprises 1 central repeat region and 2 nonrepeat end regions (N- and C-terminals). The N-terminal nonrepeat region contains a conserved region I located before the central repeat region. The C-terminal nonrepeat region contains 3 subregions, namely Th2R, conserved region II, and Th3R. Th2R and Th3R are identified as T-cell epitope regions [9] and are variable in natural *Plasmodium* populations [10]. *csp* has been studied in *P. malariae* samples collected from African countries [11]. Heterogeneity of sequences was reported among the isolates from sub-Saharan Africa with polymorphism essentially limited to the central repeat region [11]. A recent survey of *P. malariae* in Kenya also revealed high diversity in *csp* sequence [12]. However, polymorphisms in *csp* have not been reported in *P. malariae* isolates of Asia. The aim of this study is to analyse polymorphisms in *csp* of *P. malariae* field isolates collected from Thailand, Myanmar, Lao PDR, and Bangladesh. Understanding the sequence diversity within *csp* of *P. malariae* would contribute to more understanding in nature of this parasite’s distribution in the regions.

## Methods

### *Plasmodium malariae* isolates and DNA extraction

A total of 89 *P. malariae* isolates were collected from 4 different Asian countries, including Thailand, Myanmar, Lao PDR, and Bangladesh (Table 1). This study received ethical clearance from the Faculty of Tropical Medicine, Mahidol University, Thailand (MUTM2011-049-06). Genomic DNA was extracted from the isolates according to the manufacturer’s instruction (Qiagen, Germany) and stored at − 20 °C until further use.

**Table 1:** *Plasmodium* samples used in the study along with their country and year of collection

Countries	Year of collection	Species identification	Number
Thailand	2002–2008	Pm	20
Thailand	2002–2008	Pm + Pv	3
Thailand	2002–2008	Pm + Pf	1
Thailand	2002–2008	Pm + Pf + Pv	2
Thailand	2012–2016	Pm	15
Thailand	2012–2016	Pm + Pv	2
Myanmar	2009	Pm	31
Myanmar	2009	Pm + Pv	2
Myanmar	2009	Pm + Pf	5
Myanmar	2009	Pm + Pf + Pv	2
Lao PDR	2003–2010	Pm	5
Bangladesh	2008	Pm	1

### PCR amplification of *pmcsp*

The DNA samples were subjected to nested PCR [13] to confirm the presence of *P. malariae* and detect the presence of any other *Plasmodium* species. Sequences of *pmcsp* corresponding to the accession numbers S69014, U09766, J03992, AJ001525, AJ001523, AJ002582, AJ002578, AJ002580, AJ002576, AJ001526, AJ001524, AJ002583, AJ002581, AJ002577, AJ002579, AJ002575 were retrieved from NCBI (https://www.ncbi.nlm.nih.gov/). Gene-specific primers spanning the complete coding sequence of *pmcsp* were designed based on the multiple sequence alignment of *pmcsp* sequences (Table 2). Semi-nested PCR approach was used for the amplification of *pmcsp* using the conditions described in Table 2. All PCRs were carried out with 10 mM Tris–HCl (pH 8.3), 50 mM KCl, 2 mM MgCl_2_, 125 M dNTPs, 250 nM of each primer, and 4 units of *Taq* Polymerase (Kapa biosystems, USA). The PCR products were examined by gel electrophoresis. All amplified PCR products were purified using the FavorPrep™ Gel/PCR Purification Kit (Favorgen, Taiwan) and sequenced.

**Table 2:** Primer sequences and amplification conditions used for nested PCR of *pmcsp*

Primer name	Sequences (5′ to 3′)	Annealing temperature (°C)	No. of PCR cycle		Product size (bp)
			Nest 1	Nest 2	
PmCSF1	ATGAAGAAGTTATCTGTCTTAGC	50	30		1300
PmCSR1	TTAGTGAAAGAGTATTAAGACT				
PmCSF2	TTGATTTCCTCTTCCCTGGAT	52		35	1250
PmCSR1	TTAGTGAAAGAGTATTAAGACT				

### Genetic analysis of *pmcsp*

DNA sequences of *pmcsp* were read on both strands and analysed using BioEdit Sequence Alignment Editor Program as described previously [14]. DNA sequence polymorphisms, haplotype diversity, nucleotide diversity, and the rate of synonymous (dS) and nonsynonymous (dN) substitutions were calculated using DnaSP version 5.10.01 [15] and MEGA6 [16]. Phylogenetic analysis was done using the neighbour-joining method [17].

## Results

### Overall polymorphism of *pmcsp*

**Fig. 1:**
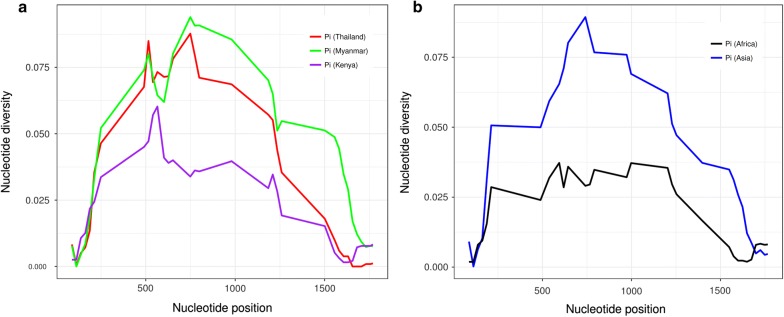
A sliding method plot with a window length of 100 bp and a step size of 25 bp using DnaSP v5 revealed a pi value in three different locations (**a**) and two continents (**b**)

**Fig. 2:**
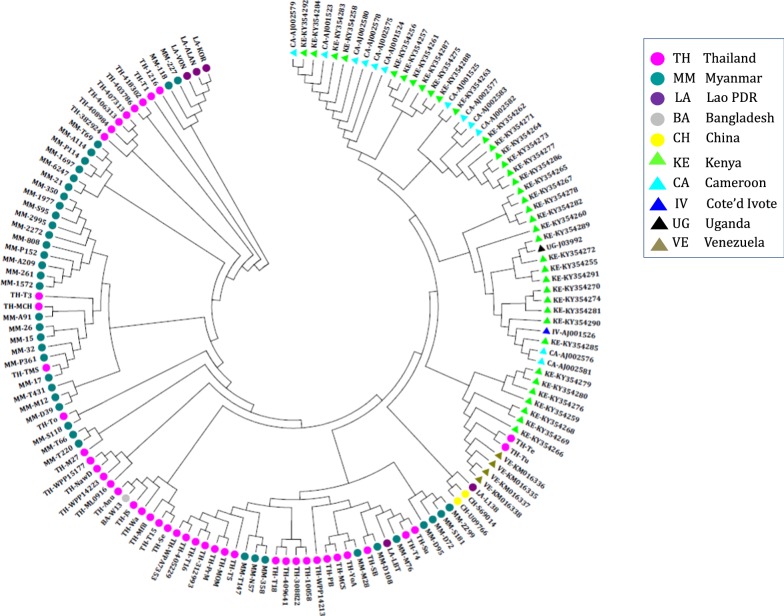
Phylogenetic analysis of 147 *Plasmodium malariae* isolates collected from different geographical regions inferred from 538 positions in the final dataset using the neighbor-joining method

*pmcsp* of 89 isolates of *P. malariae* (Thailand = 43, Myanmar = 40, Lao PDR = 5, and Bangladesh = 1; Table 1) was successfully PCR amplified and sequenced. Multiple sequence alignment of these sequences was performed, and sequences corresponding to primer-binding sites, including 12 amino acids at the 5′ end and 6 amino acids at the 3′ end, were removed. Thus, the total length of *pmcsp* used in this analysis varied from 315 to 403 amino acids. In addition to the *pmcsp* sequences obtained from the 89 isolates, 58 *pmcsp* sequences were retrieved from NCBI (https://www.ncbi.nlm.nih.gov/). All 143 *pmcsp* sequences (Asia = 91 and Africa = 52) were analyzed for DNA sequence polymorphisms. DNA divergence between populations was calculated in area containing more than 30 sequences: Thailand (n = 43), Myanmar (n = 40), and previously published results from Kenya (n = 38). Average nucleotide diversity in Kenya (pi = 0.017) was lower compared with that in Thailand (pi = 0.036) and Myanmar (pi = 0.043). When compared across different continents, the nucleotide diversity in Asia (pi = 0.042) was higher compared with that in Africa (pi = 0.015). A sliding method plot with a window length of 100 bp and a step size of 25 bp using DnaSP v5 revealed a pi value in three different locations (Fig. 1a) and two continents (Fig. 1b). The haplotype diversity of *pmcsp* was similar across these 3 countries, and ranged from 0.660 to 0.977. However, the ratio of nonsynonymous (dN) to synonymous (dS) substitutions in Kenya (dN/dS = 0.442) was lower compared with that in Thailand (dN/dS = 1.563) and Myanmar (dN/dS = 0.880). The ratio of nonsynonymous (dN) to synonymous (dS) substitutions in Africa (dN/dS = 0.411) was lower compared with that in Asia (dN/dS = 1.017) (Table 3). The neutrality test was performed in different populations though Tajima’s D, Fu and Li’s F* and Fu and Li’s D* tests. Analysis based on the non-repeat regions revealed significant differences in Asia and Africa (P  <  0.05) for Tajima’s D and Fu and Li’s F* tests (Table 3). It was indicated that these population groups have negative value reflect a lower frequency alleles than expected under a neutral model, which can result from population size expansion after a recent bottleneck or purifying selection. A total of 538 positions along *pmcsp* of all 147 *P. malariae* isolates were used for inferring their relationship with the neighbor-joining method [17]. The bootstrap consensus tree inferred from 1000 replicates which is taken to represent the evolutionary history of those 147 *P. malariae* isolates analysed. Branches corresponding to partitions reproduced in less than 50% bootstrap replicates are collapsed. Results showed that most of the *P. malariae* isolates clustered within their respective continents, with the exception of a couple of isolates from Thailand that clustered together with the four isolates from Venezuela and showed closely related to the African isolates (Fig. 2).

**Table 3:** Estimate of DNA sequence polymorphisms within nonrepeat regions of *pmcsp* gene in different populations

Population	No. of samples	Haplotype diversity	Pi	dN/dS	Fu and Li's D	Fu and Li's F	Tajima's D
Thailand	43	0.66	0.005	1.563	1.124	0.509	− 0.961
Myanmar	40	0.977	0.017	0.88	− 1.335	− 1.666	− 0.962
Kenya	38	0.925	0.007	0.442	− 3.023*	−3.138*	− 1.922*
Asia	91	0.894	0.013	1.017	− 1.904	− 2.308*	− 1.951*
Africa	52	0.906	0.006	0.411	− 4.192**	− 4.150**	− 2.235***

* P < 0.05

** P < 0.02

*** P < 0.01

### Variation in the central repeat region of *pmcsp*

*pmcsp* sequences obtained from 89 Asian isolates collected in this study were analyzed together with those previously obtained from 58 African isolates. Multiple sequence alignment of 147 *pmcsp* sequences revealed a pattern of tetrapeptide repeat units that exhibit unique characteristic for each species. The central repeat region of *pmcsp* contained the NAAG tetrapeptide repeat unit in a majority of the 147 samples, and the number of NAAG repeats varied from 0 to 79 in different samples. *P. malariae* isolates from Africa (Kenya, Cameroon) carried a higher number of NAAG repeats (42–79), whereas Asian isolates carried two range of repeat number: 0–38 repeats and 40–51 repeats (Additional file 1). The second most prevalent repeat unit was NDAG, which was present in all samples. The number of NDAG repeats varied from 2 to 9 randomly across all regions (Additional file 2). A novel tetrapeptide repeat, NAPG, was identified in this study. The number of NAPG repeats varied from 0 to 51 in samples from Thailand, Myanmar, Lao PDR, and Bangladesh (Additional file 3). Of the 43 isolates collected from Thailand, 17 isolates carried 1–40 repeats of the NAPG unit, whereas 26 isolates did not carry the NAPG repeat. Of the 40 isolates collected from Myanmar, 17 carried 11–20 NAPG repeats, 8 carried 1–10 copies, and 6 carried 21–30 copies, whereas 9 isolates did not contain the NAPG repeat. Of the 5 isolates collected from Lao PDR, 3 carried 41–60 copies of NAPG repeats and 2 did not carry the NAPG repeat. The isolate collected from Bangladesh carry 3 copies of NAPG repeat in the central repeat region.

**Fig. 3:**
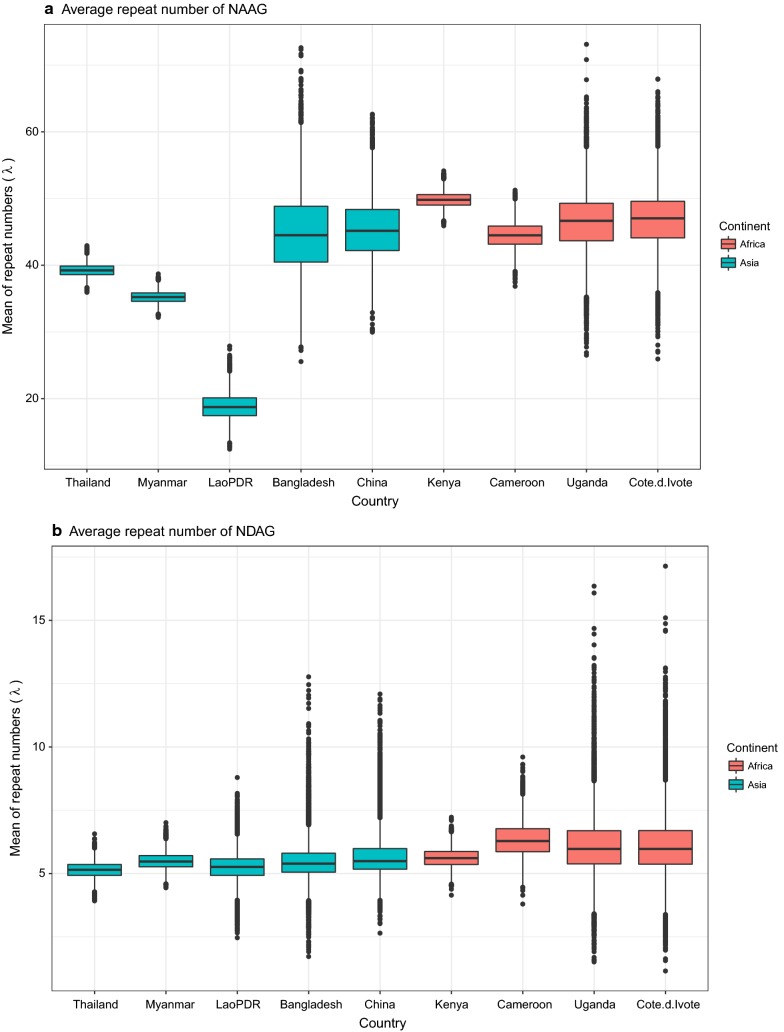
Average number of the tetrapeptide repeats: NAAG (**a**) and NDAG (**b**), between the Asian and African samples

To compare the average number of the repeat units between the Asian and African samples, the sampling model for each repeat type: NAAG and NDAG, was generated as follow;

1 $${r}_{\mathit{ij}}\sim\mathit{poiss}on({\lambda }_{\mathit{jk}})$$

2 $${\lambda }_{\mathit{jk}}\sim\mathit{normal}({\mu }_{k},{\sigma }_{k})$$

where *r*_*i*_ refer to the repeat number of the sample *i*, *j* indexes country and k indexes continent. The repeat numbers of each repeat type from the sample from any country was assumed to be sampled from a Poisson distribution where its average number was λ. These average numbers λ were thought to be varied between countries and also be distributed normally around the average numbers in the continent level (μ) with the standard deviation σ. The model was fitted to the data using the Markov Chain Monte Carlo (MCMC) technique in WinBUGS [18] (Fig. 3 and Additional file 4).

### Sequence diversity in nonrepeat regions: conserved regions I and II, Th2R, Th3R

Sequences of the nonrepeat regions of *pmcsp*, including conserved regions I and II, Th2R, and Th3R, were analyzed in 143 isolates (Table 4). Limited polymorphisms were observed in all 4 conserved regions. Seven amino acid haplotypes were identified in conserved region I. Among these, the haplotype “AVENKLKQP” was the most predominant and was identified in 82.52% of isolates (118 out of 143 isolates). In conserved region II, the haplotype “ITEEWSPCSVTCG” was identified in 93.01% of isolates (133 out of 143 isolates). The Th2R and Th3R domains showed 6 and 9 haplotypes, respectively. The most prevalent haplotypes in Th2R and Th3R were present in 91.61 and 74.83% of isolates, respectively. All polymorphisms in each Th3R haplotype were nonsynonymous. Contrary to Th2R, synonymous substitutions were found in 2 samples.

**Table 4:** Sequence polymorphism in 4 conserved domains of *pmcsp*

Haplotype	Amino acid	Number (area#)
	*Conserved region 1*	
1	PVEKKLNHP	1 (KE)
2	PVENKLKQP	1 (TH)
3	PVENKLKHP	1 (TH)
4	PFENNLNHP	1 (LA)
5	AVENKLKQP	36 (TH), 34 (KE), 30 (MM), 12 (CA), 2 (CH), 1 (LA), 1 (BA), 1 (UG), 1 (IV)
6	AVENKLKHP	10 (MM), 3 (KE), 3 (TH), 3 (LA)
7	AVENNLKQP	2 (TH)
	*Th2R*	
1	GPSEELLKNFLESIRNS	1 (MM), 1 (LA)
2	GPYEEHIKNYLESIRNS	1 (CA)
3	GPSEEHIKNIIESIRNS	1 (KE)
4	GPSEEHIKNYLKSIRNS	1 (MM)
5	GPSEEHIKNYLESIRNS	43 (TH), 37 (KE), 32 (MM) 11 (CA), 3 (LA), 2 (CH), 1 (BA), 1 (UG), 1 (IV)
6	GPSEELIKNFLESIRNS	5 (MM), 2 (TH)
	*Conserved region 2*	
1	ITEEWPPCSVTCG	6 (MM)
2	ITEKWSPCSVTCG	1 (LA), 1 (MM)
3	ITEEWSPCTVTCG	1 (LA)
4	ITEEWSPCSVTCG	43 (TH), 38 (KE), 33 (MM), 12 (CA), 3 (LA), 2 (CH), 1 (BA), 1 (IV), 1 (UG)
	*Th3R*	
1	KKVDAKNKKPAELVLSDLE	1 (MM)
2	KKVDAKNKKPAKLVLSDLE	1 (MM)
3	RKDDAKNKKPAELVLSDLE	13 (KE)
4	RKVNAKNKKPAELVLSDLE	1 (KE)
5	RKVDAKNKKPAELVLSDLE	41 (TH), 28 (MM), 21 (KE), 10 (CA), 3 (LA), 2 (CH), 1 (BA), 1 (IV)
6	RKVDAKNKKPAELVLSDVE	10 (MM), 2 (TH), 2 (LA)
7	RKVGAKNKKPAELVLSDLE	2 (KE), 1 (CA), 1 (UG)
8	RMVDAKNKKPEELVLSDLE	1 (KE)
9	REVDAKNKKPAELVLSDLE	1 (CA)

# TH, Thailand; MM, Myanmar; LA, Lao PDR; BA, Bangladesh; KE, Kenya; CH, China; CA, Cameroon; UG, Uganda; IV, Cote d’Ivote

In addition, both N- and C-terminal nonrepeat regions of *pmcsp* were used for estimating the average level of genetic differentiation between each population. The Fst [19] for all pairwise comparisons between population were significant (P < 0.001) (Table 5). The Fst value between Thailand and Myanmar (0.087) was lower than that between Thailand and Kenya (0.518) and Myanmar and Kenya (0.366). High differentiation between Asia and Africa was observed (0.404).

**Table 5:** Genetic differentiation among populations

Population 1	Population 2	Fst (P < 0.001)
Thailand (n = 43)	Myanmar (n = 40)	0.087
Thailand (n = 43)	Kenya (n = 38)	0.518
Myanmar (n = 40)	Kenya (n = 38)	0.366
Asia (n = 91)	Africa (n = 52)	0.404

## Discussion

In this study, perform genetic characterization of *pmcsp* in 89 isolates collected from Thailand, Myanmar, Lao PDR, and Bangladesh. *Plasmodium malariae* isolates collected from African countries show heterogeneity in *pmcsp* sequence [10]. Similar results have been reported for 38 *P. malariae* isolates collected from Kenya [12]. A total of 143 *pmcsp* sequences were analysed, including those obtained from 89 isolates collected in this study along with 38 previously published African isolates. The *pmcsp* DNA divergence was higher in Thailand and Myanmar compared with that in Kenya, reflecting the overall DNA divergence in Asia which was also higher than that in Africa. This finding is opposed with the divergence that have been studied in *P. falciparum csp* from Thailand and Myanmar compared to Kenya [20]. The nucleotide diversity in *pfcsp* was higher in Kenya than that in Thailand and Myanmar [20]. This is likely related to lower transmission intensity of *P. falciparum* in Thailand and Myanmar than Kenya. High diversity in *pmcsp* collected from Thailand and Myanmar might related to wide range of collecting sites in each country with various time point of sample collection (year 2002–2016). However, the overall *pmcsp* gene characteristic of the sample collected from Asia and Africa is restricted to their continents as demonstrated by the phylogenetic tree analysis. It is referred to the genetic differentiation of *pmcsp* in Asia and Africa.

The analysis focused on 2 main parts of *csp*: 1 central repeat region and 4 nonrepeat regions, including conserved regions I and II, Th2R, and Th3R. The most characteristic feature of the central repeat region in *pmcsp* is the tetrapeptide repeat unit. Previous studies have identified 2 major types of repeat units: NAAG and NDAG. The NAAG repeat was present in all *P. malariae* isolates. However, the NAAG repeat found in Asian isolates was highly polymorphic than previously reported isolates [10, 12]. *Plasmodium malariae* isolates from African countries carried high copy numbers of the NAAG repeat (40–79 copies), whereas isolates from Asian countries carried either low (0–38) or high (40–51) copy number of the NAAG repeat. By contrast, the NDAG repeat was highly conserved with low diversity, and its copy numbers varied from 2 to 9. The NDAG repeat is likely to be a universal repeat. The average numbers of the repeat units NAAG and NDAG between the Asian and African samples were used to generate the sampling model. The Markov Chain Monte Carlo (MCMC) technique in WinBUGS [18] was used to fit the data. The NAAG repeat unit revealed a wide range of repeat numbers in Asian countries compared to that of African countries. Additionally, a novel tetrapeptide repeat unit NAPG was identified in this study. More than half of the Asian isolates carried the NAPG repeat. The NAPG repeat is likely to be geographical restricted to Asian samples. To validate this hypothesis, more samples from other Asian countries should be studied. Similar repeat units have been identified in *P. falciparum* and *P. vivax*. By contrast, more than 46 repeat units have been reported in *P. knowlesi* [21]. The NANP repeat in the central repeat region of *pfcsp* has been shown to represent an important target of antibodies isolated from individuals with naturally acquired immunity to malaria [22]. The central repeat region of *csp* is the immunodominant region and each *Plasmodium* species carries a unique pattern of tetrapeptide repeats in this region. The type and copy number of tetrapeptide units may be dependent on the immune pressure in different malaria endemic regions.

Analysis of the nonrepeat regions, Th2R and Th3R, which serve as T cell epitopes revealed 6 and 9 amino acid haplotypes among 143 *P. malariae* isolates, respectively. Only one major haplotype was identified in both Th2R and Th3R, accounting for 91.61 and 74.83% of isolates, respectively. Amino acid haplotypes in Th2R and Th3R in *P. malariae* isolates were unrestricted to geographical location and showed low diversity compared with Th2R and Th3R haplotypes of *P. falciparum* [23] and *P. knowlesi* [8]. These data suggest that the central repeat region of *csp* may be responsible for the immune response in which the diversity might result in different levels of host immune system between Asia and Africa. The nonrepeat region from both N- and C-terminal parts of *pmcsp* were used to estimate genetic differentiation between populations. Pairwise comparison of *P. malariae* from Asia and Africa revealed high level of differentiation between the two continents, which is in concordance to previous studies on genetic differentiation in *P. falciparum* and *P. vivax* from Asia compared to other continents [10, 20, 24]. To gain a better understanding of the natural distribution of *pmcsp* polymorphisms, more number of samples from other malaria endemic regions should be investigated. Analysis of the genetic diversity of *pmcsp* will be valuable for understanding the population structure of *P. malariae*, which will further help in the development of strategies to eliminate malaria.

## Conclusions

This study provides valuable information on the genetic polymorphisms in *pmcsp* isolates from Asia and advances our understanding of *P. malariae* population in Asia and Africa. High genetic differentiation between Asia and Africa inferred the different population between these two continents, which might result from different host immunity in each region.

## Additional files

**Additional file 1.** Frequency distribution of the NAAG tetrapeptide repeat unit in the central repeat region of pmcsp. (a) Frequency distribution of the repeat unit in isolates collected from Thailand, Myanmar, Kenya, and Cameroon. (b) Frequency distribution of the repeat unit in isolates collected from Asia and Africa. X-axis represents the number of repeat units, and Y-axis indicates the number of samples corresponding to each repeat unit.

**Additional file 2.** Frequency distribution of the NDAG tetrapeptide repeat unit in the central repeat region of pmcsp. (a) Frequency distribution of the repeat unit in isolates collected from Thailand, Myanmar, Kenya, and Cameroon. (b) Frequency distribution of the repeat unit in isolates collected from Asia and Africa.

**Additional file 3.** NAPG tetrapeptide repeats in Plasmodium malariae field isolates from Thailand, Myanmar, Lao PDR, and Bangladesh.

**Additional file 4.** Average number of the tetrapeptide repeats: NAAG and NDAG, between the Asian and African samples (A) at the country level and (B) at the continent level.

## Abbreviation

CSP: circumsporozoite surface protein
