# Supplementary figures and images for: Genetic polymorphisms in the circumsporozoite protein of Plasmodium malariae show a geographical bias
Source: Malar J. 2018 Jul 16;17:269. doi: 10.1186/s12936-018-2413-3 (PMC6048912; doi:10.1186/s12936-018-2413-3)

## Slide 1
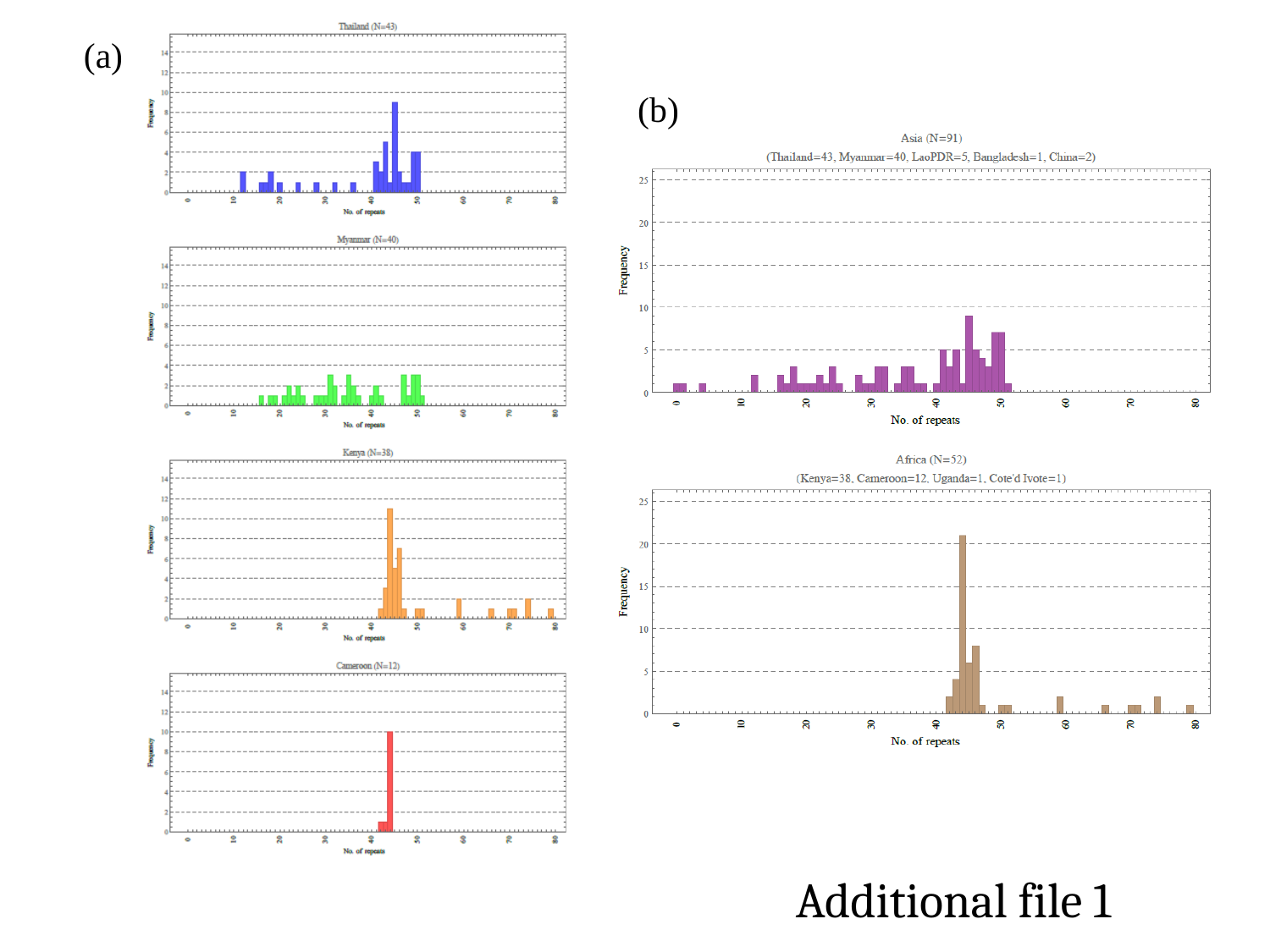

(a)
(b)
Additional file 1

Supplement: Supplementary file 1 — Additional file 1. Frequency distribution of the NAAG tetrapeptide repeat unit in the central repeat region of pmcsp. (a) Frequency distribution of the repeat unit in isolates collected from Thailand, Myanmar, Kenya, and Cameroon. (b) Frequency distribution of the repeat unit in isolates collected from Asia and Africa. X-axis represents the number of repeat units, and Y-axis indicates the number of samples corresponding to each repeat unit. [file 12936_2018_2413_MOESM1_ESM.pptx]

## Slide 1
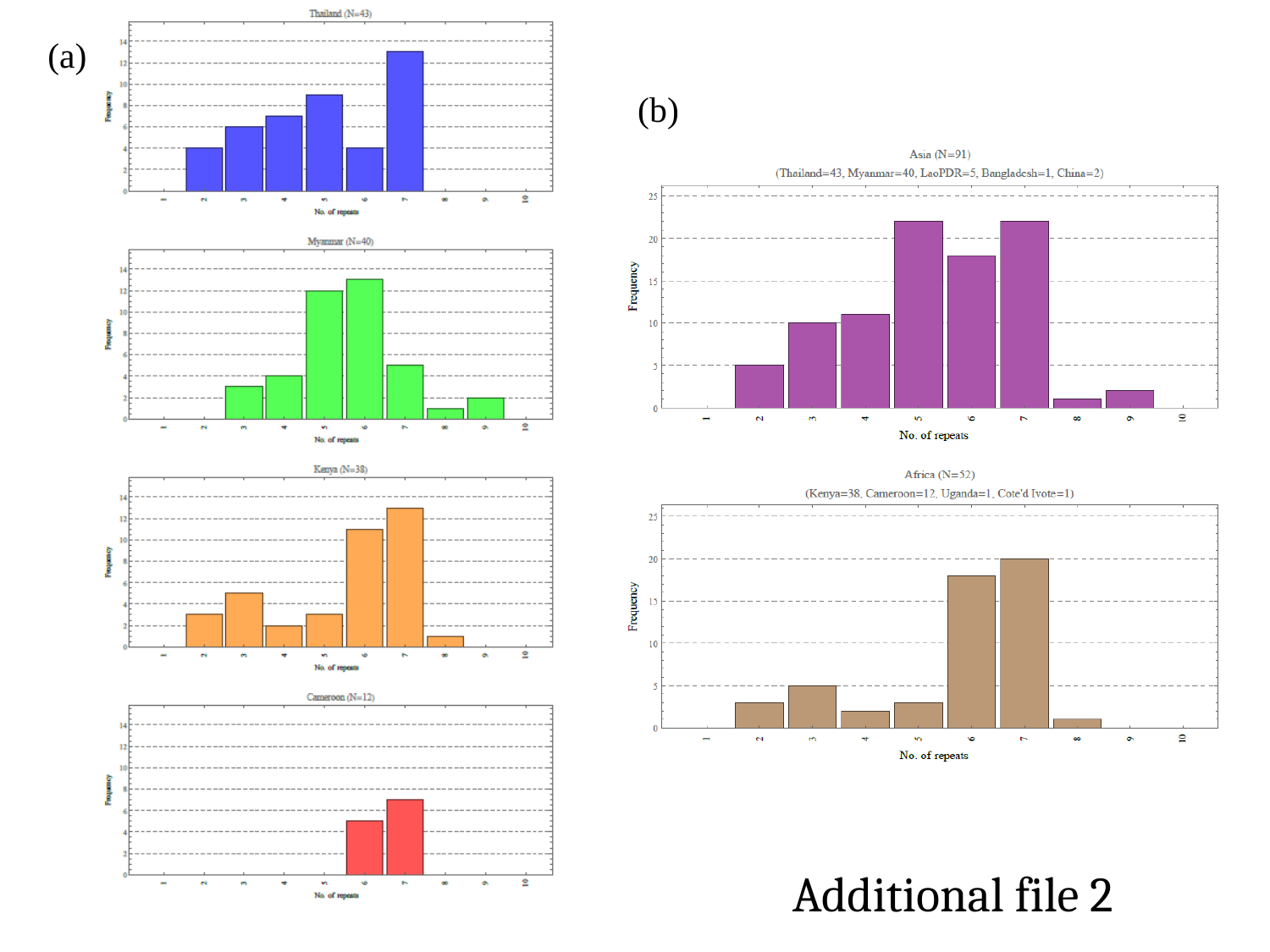

(a)
(b)
Additional file 2

Supplement: Supplementary file 2 — Additional file 2. Frequency distribution of the NDAG tetrapeptide repeat unit in the central repeat region of pmcsp. (a) Frequency distribution of the repeat unit in isolates collected from Thailand, Myanmar, Kenya, and Cameroon. (b) Frequency distribution of the repeat unit in isolates collected from Asia and Africa. [file 12936_2018_2413_MOESM2_ESM.pptx]
