# Supplementary material for: Genetic polymorphisms in the circumsporozoite protein of Plasmodium malariae show a geographical bias
Source: Malar J. 2018 Jul 16;17:269. doi: 10.1186/s12936-018-2413-3 (PMC6048912; doi:10.1186/s12936-018-2413-3)
Supplement: Supplementary file 3 — Additional file 3. NAPG tetrapeptide repeats in Plasmodium malariae field isolates from Thailand, Myanmar, Lao PDR, and Bangladesh. [file 12936_2018_2413_MOESM3_ESM.docx]

**Additional file 3.** NAPG tetrapeptide repeats in *Plasmodium malariae* field isolates from Thailand, Myanmar, Lao PDR, and Bangladesh.

| No of | Thailand | Myanmar | Lao PDR | Bangladesh |
| --- | --- | --- | --- | --- |
| NAPG repeat | (N = 43) | (N = 40) | (N = 5) | (N = 1) |
| 0 | 26 | 9 | 2 | 0 |
| 1 - 10 | 6 | 8 | 0 | 1 |
| 11 - 20 | 4 | 17 | 0 | 0 |
| 21 - 30 | 6 | 6 | 0 | 0 |
| 31 - 40 | 1 | 0 | 0 | 0 |
| 41 - 50 | 0 | 0 | 2 | 0 |
| 51 - 60 | 0 | 0 | 1 | 0 |
