# Supplementary material for: Genetic polymorphisms in the circumsporozoite protein of Plasmodium malariae show a geographical bias
Source: Malar J. 2018 Jul 16;17:269. doi: 10.1186/s12936-018-2413-3 (PMC6048912; doi:10.1186/s12936-018-2413-3)
Supplement: Supplementary file 4 — Additional file 4. Average number of the tetrapeptide repeats: NAAG and NDAG, between the Asian and African samples (A) at the country level and (B) at the continent level. [file 12936_2018_2413_MOESM4_ESM.docx]

**Additional file 4.** Average number of the tetrapeptide repeats: NAAG and NDAG, between the Asian and African samples (A) at the country level and (B) at the continent level

(A)

|  | NAAG | | | NDAG | | |
| --- | --- | --- | --- | --- | --- | --- |
| Country | median | 95% CrI | | median | 95% CrI | |
| Myanmar | 35.22 | 33.42 | 37.08 | 5.48 | 4.91 | 6.20 |
| Thailand | 39.22 | 37.41 | 41.12 | 5.15 | 4.50 | 5.76 |
| Lao PDR | 18.73 | 15.21 | 22.84 | 5.26 | 3.94 | 6.59 |
| Bangladesh | 44.50 | 33.65 | 57.72 | 5.39 | 3.99 | 7.85 |
| Cote'd Ivote | 47.02 | 37.35 | 56.51 | 5.97 | 3.72 | 9.34 |
| Cameroon | 44.48 | 40.62 | 48.46 | 6.28 | 5.17 | 7.81 |
| China | 45.16 | 36.97 | 54.88 | 5.49 | 4.40 | 8.08 |
| Uganda | 46.65 | 36.70 | 56.23 | 5.97 | 3.77 | 9.24 |
| Kenya | 49.80 | 47.56 | 52.08 | 5.61 | 4.91 | 6.38 |

(B)

|  |  | NAAG | | | NDAG | | |
| --- | --- | --- | --- | --- | --- | --- | --- |
| Parameter | Continent | median | 95% CrI | | median | 95% CrI | |
| Mean of the tetrapeptide repeat (µ) | Asia | 36.46 | 18.73 | 56.71 | 5.36 | 4.37 | 6.78 |
|  | Africa | 47.08 | 35.74 | 57.09 | 5.96 | 4.04 | 8.77 |
| Standard deviation of the tetrapeptide repeats (σ) | Asia | 14.48 | 6.72 | 49.88 | 0.48 | 0.03 | 3.13 |
|  | Africa | 5.12 | 0.99 | 28.15 | 0.98 | 0.06 | 6.49 |
